# The Cause of Angina Pectoris

**Published:** 1905-10-14

**Authors:** 


### THE CAUSE OF ANGINA PECTORIS.

In a paper on this subject Dr. Jas. Mackenzie[1] argues that as the clinical evidences of angina pectoris appear with very diverse cardiac and arterial lesions, and even in the entire absence of such lesions, the cause of these symptoms cannot be one or other form of gross pathological change, but must be some element or factor common to them all. In reviewing a series of cases suffering from anginal attacks he finds such conditions as aortic valvular disease, atheroma of the coronary arteries, myocardial degeneration (fatty and fibrous), increased arterial pressure of the elderly (often associated with atheroma and with myocardial change), and a vaso-motor spasm of the young (vaso-motor angina pectoris of Nothnagel). The problem is to find something which these possess in common, and which is also competent to produce the characteristic symptoms of angina pectoris. First, it may be observed that in all such conditions as are above noted angina pectoris is a comparatively late symptom; that is, it appears after the heart has for a prolonged period been struggling against unusual difficulties, or when the nutrition of the cardiac muscle has become seriously impaired. Again, in many instances pain is absent so long as the patient is at rest, whilst attacks of angina are generally provoked by anything that excites increased peripheral resistance (emotion, cold, arterial spasm, etc.). In short, whatever throws more work on the left ventricle causes in predisposed individuals an attack of angina pectoris. Such considerations suggest that it is in some condition of the muscular substance of the heart that the explanation of angina pectoris is to be found. That condition, whatever it be, is established when the effort at contraction meets with a relatively excessive resistance. The disproportion between contraction and resistance may be due to actual increase of the latter (as in aortic stenosis and in narrowing of the arteries), or, the resistance remaining normal, there may be failure of cardiac force from myocardial degeneration. The next step is to inquire what particular function of the cardiac muscle is at fault. From tracings made by himself Dr. Mackenzie argues that attacks of angina pectoris may occur when the excitability, conductivity, and the power to produce a rhythmical stimulus are unimpaired. He also contends that the same conclusion applies to the function of tonicity. Hence it seems necessary to admit that the particular muscular defect upon which the attacks of angina pectoris depend is in the only remaining myocardial function—namely, that of contractility. In support of this proposition three lines of evidence are advanced. First, its truth is *a priori* probable, seeing that contractility is the function that is specially strained when the heart is called upon to overcome an actual or relative increase of resistance. In the second place, the symptoms associated with, and the conditions giving rise to, the characteristic sign of depressed contractility—namely, the pulsus alternans—are of a similar nature to the symptoms in angina pectoris. And in the third place, angina pectoris may be associated with the pulsus alternans. The distinguishing feature of the pulsus alternans is the regular alternation of a strong and a weak beat, the successive waves, however, appearing at regular intervals. This alternating action of the heart is conclusive evidence of depressed contractility, though the absence of such a pulse is no proof that contractility is unimpaired. A patient may even die of failure of contractility, though the pulsus alternans has never been present. But this is certain, that all patients having a pulse of this character show a great limitation of the field of cardiac response; that is to say, while the patient is at rest the heart seems to be doing its work efficiently, but even slight exertion produces symptoms of cardiac distress. Now the pulsus alternans is apt to be provoked by the same conditions as those which excite an attack of angina pectoris. It may appear, for example, after exertion in cases of enfeebled heart, in cardiac exhaustion after prolonged high arterial tension, after paroxysmal tachycardia, etc. Further, if tracings are taken of the radial pulse in cases of angina pectoris the pulsus alternans will be found not infrequently, thus establishing once more the association of angina pectoris with exhausted contractility of the cardiac muscle. And the symptoms that accompany pulsus alternans, such as shortness of breath on exertion, general distress, and exhaustion, are manifestly of the same order as those met with in angina pectoris.

It is to be noted that the pulsus alternans is not easily detected by the finger or by listening to the cardiac sounds; it is in the sphygmographic tracing that its characters are readily displayed. Another practical point as bearing on clinical observation is that the condition of the arterial pressure is not a guide to the state of the function of contractility. Thus the pulsus alternans may be present either with an abnormally high or with a very low blood pressure.

[1] Brit. Med. Jour., October 7, 1905.

